# The orca (*Orcinus orca*) pituitary gland: an anatomical, immunohistochemical and ultrastructural analysis

**DOI:** 10.3389/fnana.2025.1626079

**Published:** 2025-07-17

**Authors:** Paula Alonso-Almorox, Alfonso Blanco, Carla Fiorito, Jose C. Gómez-Villamandos, M. A. Risalde, Javier Almunia, Antonio Fernández

**Affiliations:** ^1^Veterinary Histology and Pathology, Atlantic Center for Cetacean Research (CAIC), Veterinary School, University Institute of Animal Health and Food Safety (IUSA), University of Las Palmas de Gran Canaria, Arucas, Spain; ^2^Department of Anatomy and Comparative Pathology and Anatomy, University of Córdoba, Córdoba, Spain; ^3^Centro para el Estudio de Sistemas Marinos CESIMAR–CONICET, Puerto Madryn, Chubut, Argentina; ^4^Loro Parque Fundación, Puerto de la Cruz, Spain

**Keywords:** cetacean, orca, neuroendocrine, pituitary gland, histology, immunohistochemistry, electron microscopy, neuroendocrine system

## Abstract

The pituitary gland is central to endocrine regulation in vertebrates, coordinating key physiological processes such as growth, reproduction, and stress responses. In cetaceans, and particularly in large odontocetes like orcas (Orcinus orca), understanding pituitary structure is essential for advancing neuroendocrine research and informing welfare and health assessments. Despite their ecological, cognitive, and conservation significance, detailed morphological studies of the orca pituitary gland remain scarce. In this study, we conducted a comprehensive structural and ultrastructural analysis of the orca pituitary gland using postmortem samples from four captive individuals. We combined computed tomography, histology, immunohistochemistry, and transmission electron microscopy to examine the gland’s anatomical organization and cellular composition. Our results reveal features consistent with other cetaceans as well as species-specific characteristics, including the distribution and morphology of endocrine cells within the adenohypophysis and neurohypophysis. These findings provide the first integrated anatomical and ultrastructural reference for the orca pituitary gland, offering valuable insights into cetacean neuroendocrinology and supporting improved species-specific welfare evaluation, health monitoring, and management practices for orcas under human care.

## 1 Introduction

The pituitary gland plays a fundamental role in vertebrate neuroendocrinology, regulating essential physiological functions such as growth, metabolism, reproduction, and stress responses through the hypothalamic-pituitary axis. Despite its critical function, comprehensive anatomical and ultrastructural studies of the pituitary gland in cetaceans remain scarce, with most available data derived from recent studies on small odontocete species such as the bottlenose dolphin (*Tursiops truncatus*) and common dolphin (*Delphinus delphis*) (Vuković et al., 2011; Cozzi et al., 2016; Alonso-Almorox et al., 2025). Although earlier brief descriptions of the pituitary gland exist for mysticete species and larger odontocetes such as the beluga whale (*Delphinapterus leucas*) (Wislocki and Geiling, 1936; Yablokov et al., 1972), they are generally outdated and lack the anatomical and histological detail required for comparative neuroendocrinological research. Moreover, interspecies differences in pituitary morphology and function may exist among cetaceans, driven by adaptations to distinct ecological niches, diving behavior, and metabolic demands.

Orcas (*Orcinus orca*) are among the most widely distributed cetaceans, inhabiting diverse marine ecosystems from polar to tropical waters. Genetic and ecological studies suggest that orcas comprise multiple ecotypes—and possibly even distinct subspecies—each adapted to specific environmental conditions (Morin et al., 2024). The term “orca” instead of “killer whale” when referring to this species is used to avoid any negative connotations associated with the latter (Gregg et al., 2020). Adopting a more neutral terminology is not only a matter of scientific accuracy but also an essential step toward fostering broader public support for conservation. As suggested by recent research, conservation outcomes can be influenced by the language used to describe species, particularly those perceived as controversial or emotionally charged (Stevens et al., 2014; Thommen et al., 2021). Despite their public relevance, ecological significance, complex social structures, and physiological adaptations, many aspects of this species’ basic anatomy, including the neuroendocrine system, remain largely unexplored (Ford, 2009; de Bruyn et al., 2013; Johnson, 2018). This gap in anatomical knowledge limits our understanding of their physiology and health, and hinders conservation efforts aimed at preserving distinct populations. As apex predators, orcas play a pivotal role in maintaining the balance of marine ecosystems, and are widely recognized as keystone, and flagship species in conservation efforts (Kau et al., 2025). Yet they are increasingly threatened by a combination of natural and anthropogenic stressors—including pollution, prey depletion, noise disturbance, and habitat degradation—which may impair their health and long-term viability (Lacy et al., 2017; Williams et al., 2024). In this context, advancing our understanding of their physiology is essential to inform conservation strategies and assess population resilience.

On the other hand, the orca captive industry has increasingly attracted both public and scientific attention since its start in the early 1960s, when the first successful captures were performed (Olesiuk et al., 1990; Pollard, 2014). As the social, political and scientific landscape has evolved, and animal welfare has become an highly prioritized concept, the evaluation of health and welfare of captive species has become a critically important, particularly amid growing ethical debates regarding the wellbeing of captive cetaceans (Lott et al., 2017; Lauderdale et al., 2021). In this context, there is a growing emphasis on identifying bioindicators that objectively assess welfare status, integrating physiological, behavioral, and endocrinological measures (Manteca et al., 2016). In recent years, several endocrine parameters, such as corticosteroids, have been evaluated as welfare and stress indicators in captive cetaceans, including orcas (Suzuki et al., 1998, 2003; Steinman et al., 2020; Steinman and Robeck, 2021; Lemos et al., 2024).

Although most of these indicators have been developed within captive settings, they hold potential for application to wild populations. Chronic stress has been implicated in various health issues in wild marine mammals, including immune suppression and reproductive dysfunction in orcas (Krahn et al., 2009). Thus, understanding the neuroendocrine system is crucial for the health evaluation of both captive and wild individuals.

Central to the neuroendocrine system, the hypothalamic-pituitary-adrenal (HPA) axis plays a pivotal role in coordinating physiological responses to stress. The pituitary gland, through the secretion of adrenocorticotropic hormone (ACTH) by the adenohypophysis, stimulates the adrenal cortex to release glucocorticoids, which modulate energy metabolism, immune function, and behavioral responses to environmental challenges (Tsigos and Chrousos, 2002). Assessing the functionality and potential alterations of the HPA axis in cetaceans, particularly under human care, is critical for understanding physiological stress responses and welfare outcomes (Karaer et al., 2023).

Previous studies in delphinids, mostly those in common dolphins (*Delphinus delphis*) and common bottlenose dolphins (*Tursiops truncatus*), have provided detailed histological, immunohistochemical, and ultrastructural characterizations of the pituitary gland, identifying key hormone-secreting cell types and their distribution across the adenohypophysis and neurohypophysis (Wislocki, 1929; Cowan et al., 2008; Vuković et al., 2011; Alonso-Almorox et al., 2025). Building upon these findings, the present study provides the first structural and ultrastructural description of the pituitary gland in the orca (*Orcinus orca*). By investigating the cellular composition and structural organization of the pituitary, we aim to gain insights into species-specific adaptations, endocrine regulation, and potential welfare concerns in both captive and wild settings.

This research aims to expand our understanding of cetacean neuroendocrinology by offering novel comparative data on the orca pituitary gland. Through the integration of histological, immunohistochemical, and imaging techniques, we seek to contribute to the broader field of marine mammal neuroendocrinology and provide valuable reference data for future investigations into cetacean health, welfare, physiology, and conservation.

## 2 Materials and methods

### 2.1 Animals

We collected the pituitary glands from four orcas (*Orcinus orca*) that died between 2021 and 2025 and had been born under human care at Loro Parque (Canary Islands, Spain). The sample included three females (two adults and one juvenile) and one adult male. Full post-mortem examinations were conducted within 12 hours post-mortem, following the standardized protocol described by Kuiken and García-Hartmann (1991), incorporating specific adaptations implemented by the pathology team at the Institute for Animal Health and Food Safety, Universidad de Las Palmas de Gran Canaria (IUSA, ULPGC) (Arbelo et al., 2013; Díaz-Delgado et al., 2018). These were performed by the veterinary pathology team (IUSA) at the Faculty of Veterinary Medicine, ULPGC. At the time of necropsy, all individuals were classified as either “very fresh” or “fresh,” according to the categories established by Kuiken and García-Hartmann (1991). The individual characteristics of the animals, including sex, age class, and cause of death, are summarized in Table 1.

**TABLE 1:** Individual information for the four bred in managed care orcas (*Orcinus orca)* specimens included in this study.

Name	Sex	Age	Origin	Date of death	Necropsy date	Cause of death
Ula	Female	3 years	Captive	08/10/2021	09/10/2021	Intestinal volvulus. Endotoxic shock.
Skyla	Female	17 years	Captive	03/12/2021	03/12/2021	Intestinal volvulus. Endotoxic shock.
Kohana	Female	20 years	Captive	09/14/2022	09/14/2022	Cardiopulmonary chronic disease. Open patent ductus arteriosus.
Keto	Male	29 years	Captive	22/11/2024	22/11/2024	Severe bacterial pneumonia. Septic/endotoxic shock.

Data include name, sex, age, origin, date of death, necropsy date, and diagnosed cause of death based on full post-mortem examination.

Anatomical extraction of the pituitary gland was performed following the protocol established by Alonso-Almorox et al. (2025). The glands were subsequently processed for histological, ultrastructural, and immunohistochemical analyses using optical microscopy, transmission electron microscopy (TEM), and immunostaining techniques. Additionally, a computed tomography (CT) scan was performed on the head of the juvenile female prior to dissection to assess the precise anatomical location and structural characteristics of the pituitary gland.

### 2.2 Computed tomography imaging (CT) study

The head of a juvenile female orca was dissected and scanned using a 16-slice helical CT scanner (Toshiba Astelion, Canon Medical System, Tokyo, Japan) at the Veterinary Hospital HCV (Hospital Clínico Veterinario) of the Universidad de Las Palmas de Gran Canaria (ULPGC), acquiring transverse CT sequential images. For fitting reasons, only the head was placed inside the scanner. The head was positioned in dorsoventral position during the scan. The acquired images had a slice thickness of 1 mm wish a slice increment of 0.80 mm. We used a variety of window settings by adjusting the window widths (WWs), and window levels (WLs): a bone window setting (WW = 2,700; WL = 550), a soft tissue window setting (WW = 500; WL = 50). The scan was conducted at 135 kilovolt peak (kVp) and an exposure of 75 mAs. The gantry tilt was set to 0°. The analysis was performed with OsiriX MD© software (Pixmeo, Geneva, Switzerland).

### 2.3 Histochemical analysis

For histological evaluation, pituitary samples were embedded in paraffin and sectioned into 5 μm slices. Tissue sections were then stained using hematoxylin and eosin (HE), periodic acid-Schiff–orange G (PAS-OG), Masson’s trichrome (MT), Masson’s trichrome–orange G (MT-OG) and Luxol fast blue stain (LFB). All staining reagents were sourced from Panreac Applichem (Barcelona, Spain) or VWR Chemicals (Radnor, PA, USA). Microscopic imaging was carried out either using an Olympus BX51 microscope, equipped with a DP21 camera and a 0.5X (U-TV0.5XC-3) adapter (Olympus Corp., Tokyo, Japan); or with a Motic Easy Scan One slide scanner (Motic, Hong Kong, China) and the Motic DSAssistant© Software (Motic, Hong Kong, China).

### 2.4 Immunohistochemical analysis

Formalin-fixed, paraffin-embedded (FFPE) pituitary sections (3 μm thick) were placed on Vectabond^®^-treated slides (Vector Laboratories, Newark, CA, USA) before undergoing immunohistochemical processing. The following primary antibodies were used: polyclonal anti-ACTH antibody (206A-74) at a 1:75 dilution (Merck, Darmstadt, Germany), polyclonal anti-alpha-MSH antibody (M0939-0.2ml) at a 1:500 dilution (Merck, Darmstadt, Germany), and polyclonal anti-TSH antibody (211A-14) at a 1:100 dilution (Merck, Darmstadt, Germany). Standardized protocols were followed to ensure accuracy.

To evaluate the suitability of commercially available antibodies for use in orcas, we performed *in silico* validation using BLASTp analysis (NCBI Protein-Protein BLAST) to compare the amino acid sequences of the immunogenic regions targeted in the host species (*Homo sapiens*) with the corresponding *O. orca* protein sequences. For each antibody, we retrieved the target protein sequence from the UniProt or NCBI database, identified the epitope or immunogenic region when available, and aligned it with the orthologous orca sequence using BLASTp. Sequence similarity was assessed based on percent identity and query coverage. All three primary antibodies used (anti-ACTH, anti-MSH, and anti-TSH) showed high similarity with their respective orca targets, with 100% identity for POMC-based epitopes (ACTH and MSH) and 89.13% identity for TSH. These results suggest strong to likely cross-reactivity with orca tissue (Supplementary Table 1), supporting their application in this study.

The antibodies and immunohistochemical conditions used in this study are detailed in Table 2. Positive controls consisted of common bottlenose dolphin pituitary tissue, while negative controls were prepared by replacing the primary antibody with non-immune homologous 10% serum in phosphate-buffered saline (PBS) or by using tissues that did not express the target antigen.

**TABLE 2:** Summary of primary antibodies and immunohistochemical conditions used for the detection of ACTH, MSH, and TSH in the pituitary glands of orcas (*Orcinus orca*).

Target hormone	Primary antibody	Antibody type	Catalog N°	Host species	Dilution	Antigen retrieval	Incubation conditions	Detection system and chromogen
ACTH	Anti-ACTH	Polyclonal	(Cell Marque Cat# 206A-74, RRID:AB_1158079)	Rabbit	1:75	Protease digestion (0.1%, PBS)	Overnight at 4°C	Vectastain Elite ABC Kit DAB
MSH	Anti-α-MSH	Polyclonal	(Sigma-Aldrich Cat# M0939, RRID:AB_260462)	Rabbit	1:500	Heat-induced (citrate buffer, pH 6.0, 96°C)	Overnight at 4°C	Vectastain Elite ABC Kit DAB
TSH	Anti-TSHβ	Polyclonal	(Cell Marque Cat# 211A-1)	Rabbit	1:100	Heat-induced (citrate buffer, pH 6.0, 96°C)	Overnight at 4°C	Vectastain Elite ABC Kit DAB

The table includes the antibody type, manufacturer, catalog number and research resource identifier (RRID), dilution, antigen retrieval method, incubation conditions, and detection system.

Immunostaining was performed using 3,3’-diaminobenzidine (DAB) as the chromogen. Tissue sections were first deparaffinized in xylene and progressively rehydrated in ethanol solutions. To inhibit endogenous peroxidase activity, sections were treated with 3% hydrogen peroxide in methanol for 30 min at room temperature. Antigen retrieval was carried out using either heat-induced epitope retrieval in citrate buffer (pH 6.0) at 96°C for 15 min or enzymatic digestion with 0.1% protease from Streptomyces (P5147) (Merck, Darmstadt, Germany) in PBS. Samples were incubated with primary antibodies at 4°C overnight, followed by a 30-min incubation with a biotinylated secondary antibody at room temperature. Detection was achieved using the VECTASTAIN^®^ Elite ABC-Peroxidase Kit (PK-6100) (Vector Laboratories, Newark, CA, USA), with DAB development lasting between 2 and 7 min. Mayer’s hematoxylin was applied for counterstaining (5–10 min), and slides were mounted using Faramount Aqueous Medium (S3025) (Agilent, Santa Clara, CA, USA).

### 2.5 Ultrastructural analysis

For ultrastructural examination, rectangular sections of the adenohypophysis, measuring approximately 2 × 5 mm with a thickness of less than 1 mm, were carefully excised from a sagittal plane using a precision blade. The study did not include samples from the neurohypophysis, as the analysis focused on the endocrine components of the pituitary gland.

Tissue samples were rinsed in PBS for 10 min, followed by overnight fixation at 4°C in a 2.5% glutaraldehyde solution prepared in 0.1 M phosphate buffer (pH 7.4). Post-fixation was carried out in 1% osmium tetroxide (Merck, Darmstadt, Germany) in the same phosphate buffer for 30 min. After dehydration through a graded ethanol series, samples were embedded in Araldite resin M (10951) (Merck, Darmstadt, Germany). Semi-thin sections were prepared using an LKB ultramicrotome and stained with toluidine blue, while ultrathin sections underwent staining with uranyl acetate and lead citrate. Transmission electron microscopy (TEM) imaging was performed with a JEM 1400 (JEOL, Ltd.) at the Central Microscopy Research Facilities, Universidad de Córdoba (Spain).

The results below summarize the structural and cellular features of the orca pituitary gland as revealed by our multimodal investigation.

## 3 Results

### 3.1 CT study

**FIGURE 1:**
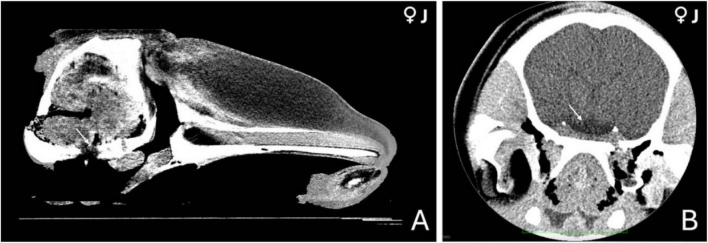
Computed tomography (CT) images of the head of a 3-year-old female orca (*Orcinus orca*). **(A)** Mid-sagittal section showing the anatomical region where the pituitary gland is located at the base of the skull (indicated by a white arrow). **(B)** Coronal section at the level of the hypothalamus, highlighting a hypodense area (white arrow) consistent with a highly vascularized region, potentially corresponding to the hypophyseal vascular pad.

Due to the limited soft tissue contrast of computed tomography, direct visualization of the pituitary gland was not possible. However, by orienting the scan to the midbrain region—where the hypophysis is anatomically expected—we were able to approximate its location. At this level, within the central base of the skull, a hypodense area was observed, consistent with the vascularized tissue that typically surrounds the gland (Figures 1A, B).

Although no definitive margins of the hypophysis could be discerned, this low-density region appeared embedded within a subtle concavity of the sphenoid bone (Figure 1A), likely representing the shallow depression of the sphenoid bone where the pituitary gland sits. This indirect evidence, along with anatomical positioning, provided the best estimate of the gland’s location in the CT images.

### 3.2 Macroscopic and histological evaluation of the pituitary gland

**FIGURE 2:**
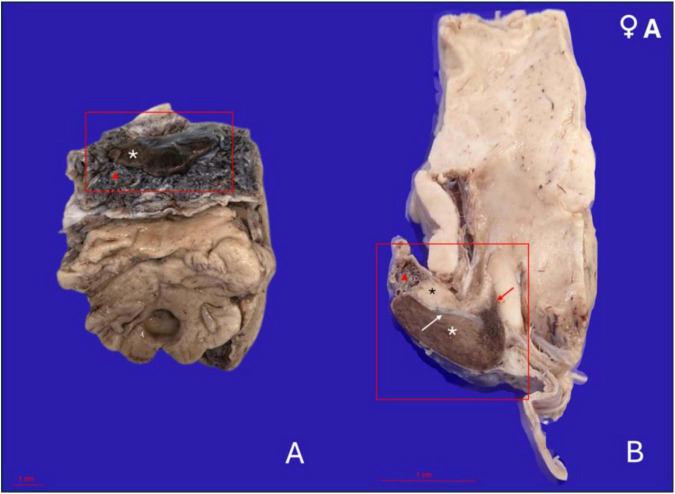
Gross anatomical features of the pituitary gland in a 17-year-old female orca (*Orcinus orca*) following removal from the brain and formalin fixation. **(A)** Caudoventral perspective of the pituitary gland *in situ*, still attached to a portion of the brain maintained for anatomical orientation. The adenohypophysis appears as a broad, flattened, bean-shaped structure with a dark brown coloration (white asterisk). It is embedded within a thick, highly vascularized pad (red triangle). **(B)** Midsagittal section of the pituitary gland showing the full extent of the hypophysis and its connection to the hypothalamus. The adenohypophysis (white asterisk) is larger and located ventrally, while the neurohypophysis (black asterisk) is situated dorsally and caudally, with a paler whitish hue. The gland is surrounded by the fibrous and highly vascularized pad (red triangle). The infundibulum (red arrow) connects the hypothalamus to the neurohypophysis, representing the neuroendocrine interface. The fibrous dural recess separating both hypophyseal lobes is also visible (white arrow).

In all four orcas examined, the pituitary gland presented as a flat, bean-shaped structure, broad and elongated, yet notably thin in its dorsoventral profile. Its length consistently exceeded its height by approximately a factor of two. As the glands were extracted in their anatomical relation, still attached to the ventral aspect of the brain, the adenohypophysis was the predominant portion visible on gross examination, appearing considerably larger than the neurohypophysis (Figure 2A).

The gland was embedded within a dense, fibrous pad that was remarkably thick and highly vascularized, enveloping the entire pituitary region. This pad contained a rich and intricate network of arteries and veins, arranged in a branching and interlacing pattern (Figure 2A). This vascular plexus followed the organization pattern and morphology of the *rete mirabile*. On gross examination, the vascular meshwork extended dorsally and laterally around the gland, forming a prominent connective and vascular interface between the hypophysis and the surrounding cranial structures.

Upon performing a mid-sagittal section through the gland, the neurohypophysis became apparent, located dorsally and slightly caudally relative to the central region of the adenohypophysis. It was approximately three times smaller in volume than the adenohypophysis and was clearly delineated by a fibrous band of tissue, which then continued to form a partial capsule around the larger adenohypophyseal lobe (Figure 2B).

In terms of coloration, the neurohypophysis exhibited a pale, whitish to yellowish hue, consistent with its neural composition. In contrast, the adenohypophysis displayed a deeper brownish-wine coloration, which intensified around the area corresponding to the *pars tuberalis* and infundibulum, likely due to the increased vascularization of this region.

**FIGURE 3:**
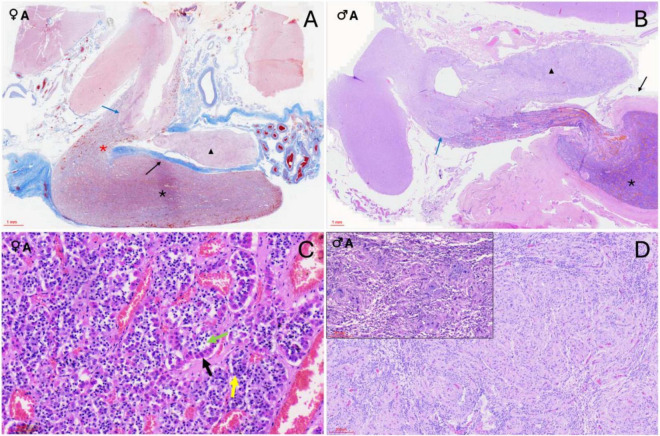
Histological sections of orca (*Orcinus orca*) pituitary glands. **(A)** Masson’s trichrome-stained submacroscopic section of a 17-year-old female orca pituitary gland showing both the neurohypophysis (black triangle) and the adenohypophysis in its *pars distalis* (black asterisk), which continues as the *pars tuberalis* at the level of the infundibular stalk (red asterisk). A clearly demarcated digitiform neuroendocrine connection (blue arrow) links the endocrine and nervous tissues. The connective dural recess separating both lobes is also visible (black arrow). **(B)** Hematoxylin–Eosin (H–E)–stained section of a 29-year-old male showing the same structures: neurohypophysis (black triangle), adenohypophysis with *pars distalis* (black asterisk) and *pars tuberalis* (white asterisk), neuroendocrine interface (blue arrow), and dural recess (black arrow). **(C)** H–E staining (40×) of the adenohypophysis in an adult female, showing acidophils (black arrow), basophils (yellow arrow), and chromophobes (green arrow) arranged in cords or clusters. **(D)** H–E staining (10×) of the neurohypophysis in an adult male. The inset shows a Luxol fast blue stain of the same section (10×), revealing the unmyelinated axons, pituicytes, and capillaries.

**FIGURE 4:**
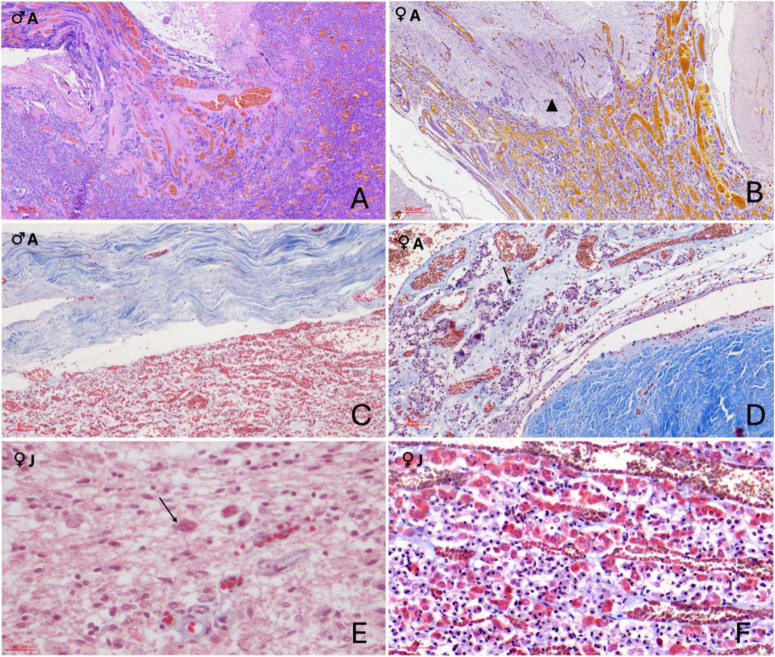
Histochemical sections of the orca (*Orcinus orca*) pituitary gland. **(A)** Hematoxylin–Eosin (H–E) staining (10×) of the *pars tuberalis* in an adult male, showing its highly vascularized structure; **(B)** Periodic acid–Schiff–Orange G (PAS–OG) staining (3×) of the neuroendocrine connection in an adult female, highlighting the intricate and delicate intertwining of endocrine and nervous tissues (black triangle) at the level of the infundibulum. This area displays intense vascularization. **(C)** Masson’s trichrome–Orange G (TCM–OG) staining (60×) of the dorsal adenohypophysis in an adult male, showing the connective dural recess separating the adenohypophysis and neurohypophysis.; **(D)** Masson’s trichrome–OG staining (15×) of the *pars tuberalis* in an adult female. The image reveals thick connective tissue enveloping the gland and its extension toward the hypothalamus. The slender, yet highly vascularized, tuberal portion contains rosette-like endocrine cell arrangements between capillaries, as well as intensely red-stained extracellular material (black arrow) suggestive of protein-rich hormonal storage or byproducts; **(E)** TCM–OG staining (60×) of the neurohypophysis in a juvenile female. Nerve fibers, capillaries, pituicytes, and granule aggregations compatible with Herring bodies (black arrows) are observed. **(F)** Masson’s trichrome–OG staining (30×) of the adenohypophysis in a juvenile female. Chords of acidophils, basophils, and chromophobes are closely associated with capillaries, and thin connective tissue fibers are distinguishable within the parenchyma due to the trichrome stain.

Histological examination revealed a well-preserved gland with clearly distinguishable adenohypophyseal and neurohypophyseal regions. The entire structure was enveloped by a dense connective tissue capsule containing a complex and highly vascularized retiform architecture. Numerous thin-walled blood vessels were observed running in parallel and perpendicular orientations, branching and converging within the fibrous tissue surrounding the gland (Figures 3A, B). This vascular mesh extended into the glandular parenchyma, particularly at the dorsal and lateral boundaries of the adenohypophysis, and was especially prominent at the interface between the two hypophyseal lobes. At this junction, the rete-like structure formed a thick dural recess, acting as a distinct anatomical boundary between the lobes (Figure 4C).

The adenohypophysis was organized as epithelial hormone-secreting cells arranged in cords, clusters, and anastomosing ribbons, supported by a prominent capillary network and interstitial stroma (Figure 3C). Within the *pars distalis*, cells were densely packed and showed differential staining affinities in hematoxylin and eosin (H&E), corresponding to chromophilic (acidophilic and basophilic) and chromophobic types. Histochemical staining further differentiated cell populations: PAS–Orange G (PAS-OG) highlighted glycoprotein-rich basophilic cells, particularly those in the central and rostral *pars distalis*; trichrome Masson (TCM) and TCM–OG staining accentuated the connective tissue framework, capillary distribution, and interstitial stroma, aiding in the delineation of endocrine cell cords and vascular associations (Figures 4D, F). The *pars tuberalis* was composed of elongated cords of endocrine cells in close association with blood vessels. The infundibular stalk appeared particularly long and slender in this species, owing to the extended *pars distalis*, and was flanked by connective tissue that maintained the gland’s integrity up to its junction with the hypothalamus (Figures 3A, B, 4A, B).

The neurohypophysis was composed of loosely arranged fibrous tissue, with low cellular density. It contained unmyelinated axons interspersed with glial-like pituicytes and a dense network of capillaries (Supplementary Figure 1 and Figure 3D). PAS-positive eosinophilic accumulations, consistent with neurosecretory material (Herring bodies), were observed in one of the female adult samples (Figure 4E). At the junction with the infundibulum, a distinct but intimate interface was observed, where nervous and endocrine tissues were tightly interwoven in a digitiform manner—finger-like projections of each tissue type interlacing with the other, yet remaining histologically distinct (Figure 4B). This arrangement suggested a structurally complex but clearly demarcated neuroendocrine interface in the orca.

### 3.3 Immunohistochemical evaluation

Immunolabelling of the adenohypophysis in orcas revealed specific distribution patterns for ACTH, MSH, and TSH, consistent with a structurally and functionally organized gland.

#### 3.3.1 ACTH expression

**FIGURE 5:**
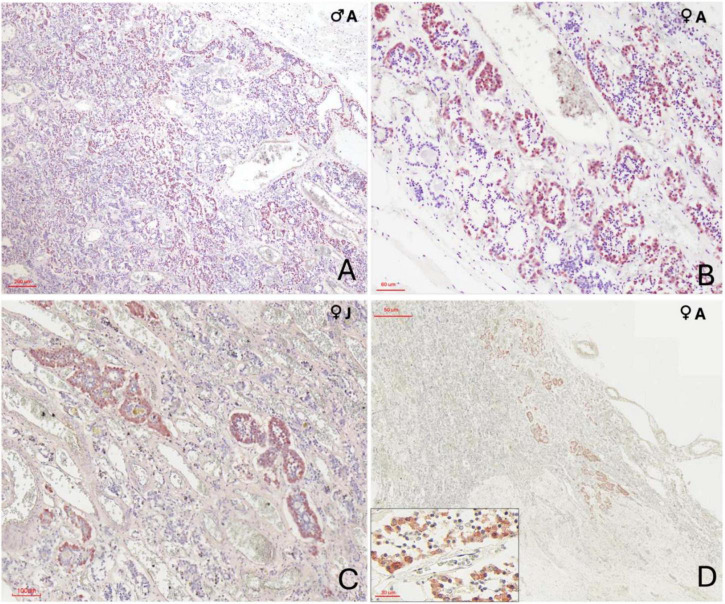
Immunohistochemical characterization of adenohypophyseal cell populations in orcas using anti–adrenocorticotropic hormone (ACTH) antibody. **(A)** Adult male, 10× magnification. ACTH-positive cells display strong cytoplasmic staining and appear either individually dispersed or organized in small rosettes throughout the *pars tuberalis* parenchyma; **(B)** Adult female, 40× magnification of the *pars tuberalis*. ACTH-positive cells show clear cytoplasmic labeling and are arranged in rosettes closely associated with capillaries; **(C)** Juvenile female, 20× magnification at the transition between the *pars distalis* and *pars tuberalis*. Cells exhibit strong cytoplasmic staining and a clean rosette-like arrangement surrounding capillaries; **(D)** Adult female, 2× magnification at the terminal end of the infundibulum in the neuroendocrine connection. ACTH-positive cells, though fewer, maintain a rosette-like organization and show clear cytoplasmic staining. Labeling occurs at the distal region of the endocrine tissue, in close anatomical association with the nervous tissue digitations. The inset provides at higher magnification (40×) the clear visualization of the polygonal morphology of ACTH-positive cells, highlighting their distinct cytoplasmic boundaries and organization.

Cells immunoreactive for ACTH were the most numerous across all specimens, showing robust cytoplasmic staining. These cells were densely distributed within the pars distalis, where they either formed extensive, interconnected clusters, ot appeared dispersed throughout the parenchyma (Figure 5A). Moreover, these cells were also concentrated in the pars tuberalis, often forming rosette-like structures aligned parallel to vascular channels (Figures 5B, C, D). ACTH-positive cells were characterized by a large cytoplasmic volume and moderately polygonal profiles. The male orca exhibited a noticeably higher abundance of ACTH-labeled cells.

#### 3.3.2 MSH expression

**FIGURE 6:**
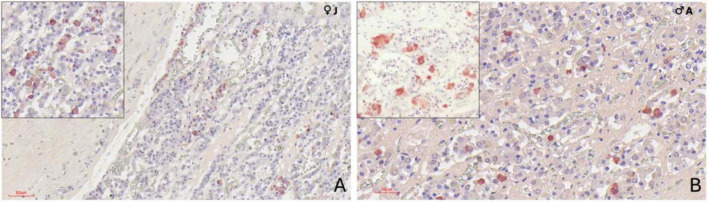
Immunohistochemical (IHC) staining of orca (*Orcinus orca*) adenohypophysis. **(A)** IHC staining using anti–melanocyte-stimulating hormone (MSH) antibody in a juvenile female orca at 40× magnification. MSH-positive cells exhibit distinct cytoplasmic labeling, appearing predominantly as single cells, and occasionally in pairs or small clusters. These cells are more concentrated in the dorsal region of the *pars distalis*, near the dural connective recess, but are also scattered throughout the *pars tuberalis*. The inset (40×) highlights scattered MSH-positive cells in the parenchyma; **(B)** IHC staining for thyroid-stimulating hormone (TSH) in an adult male orca at 40× magnification. TSH-positive cells appear as small, polygonal cells with strong, well-defined cytoplasmic staining, dispersed individually across the adenohypophysis. The inset provides a higher-contrast view (40×, with reduced hematoxylin counterstaining) emphasizing cytoplasmic immunolabelling.

MSH-positive cells were observed throughout the adenohypophysis but were more frequently found within the dorsal ridge of the pars distalis, arranged either in small clusters of two or three cells or individually dispersed. In the pars tuberalis, MSH-immunoreactive cells were less densely distributed and appeared more isolated. Staining intensity was generally moderate, with clear cytoplasmic localization (Figure 6A).

#### 3.3.3 TSH expression

TSH immunoreactivity was less widespread than that of ACTH and MSH (Supplementary Figures 2, 3). Positive cells typically occurred as single units or in small pairs of two cells. These were primarily situated in the pars tuberalis, with sporadic presence in the pars distalis. TSH-positive cells exhibited a compact morphology, with a small and polygonal shape, and strong cytoplasmic labeling (Figure 6B).

### 3.4 Ultrastructural evaluation (TEM) of the adenohypophysis

**FIGURE 7:**
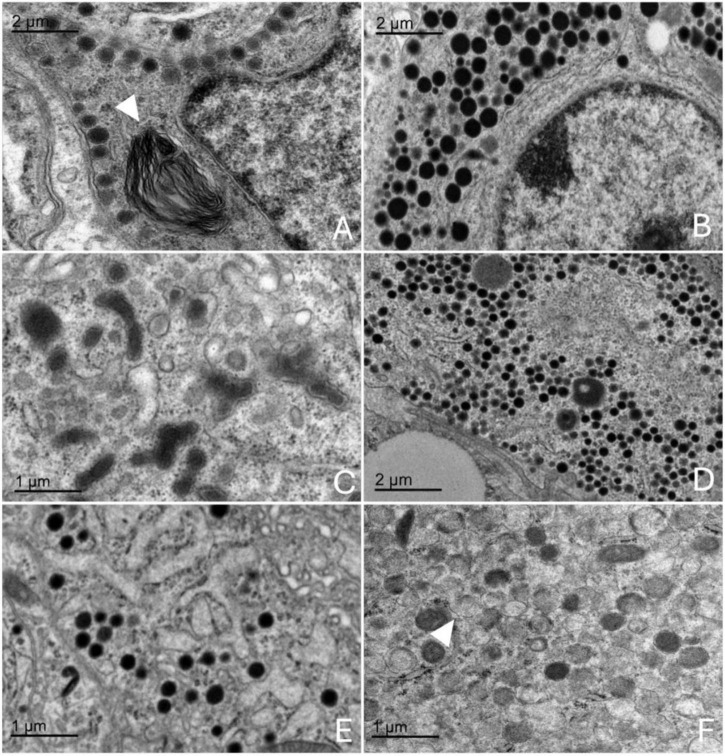
Transmission electron microscopy images of the adenohypophysis of the orca (*Orcinus orca*), showing the ultrastructural features of the different endocrine cell types. **(A)** Corticotroph, characterized by small, spherical electron-dense granules aligned near the plasma membrane, a myelin figure can be observed within the cytoplasm (white triangle) **(B)** Somatotroph, showing densely packed, medium-to-large granules concentrated toward one pole of the cytoplasm **(C)** Lactotroph, with pleomorphic secretory granules of varying sizes and densities scattered throughout the cytoplasm. **(D)** Gonadotroph, displaying medium-sized granules distributed evenly across the cytoplasm. **(E)** Thyrotroph, with small, densely packed granules and compact cytoplasmic organization. **(F)** Melanotroph, distinguished by granules with variable electron density and irregular or scalloped membranes (white triangle).

**FIGURE 8:**
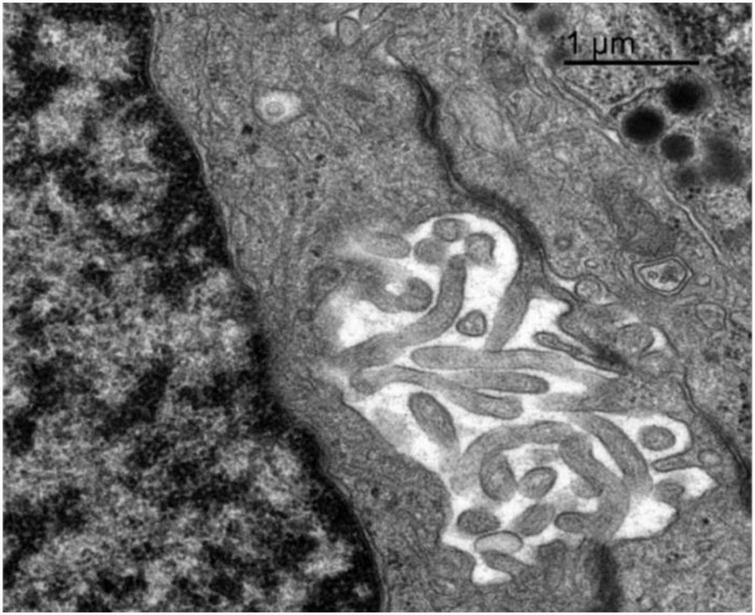
Transmission electron microscopy image of the adenohypophysis of the orca (*Orcinus orca*), showing the ultrastructural features of a follicular cell. This non-secretory, non-granular cell type is observed lining a follicular cavity, which contains numerous villi-like cytoplasmic projections extending into the lumen. These projections suggest a potential role in intercellular communication or regulation of the follicular microenvironment.

**FIGURE 9:**
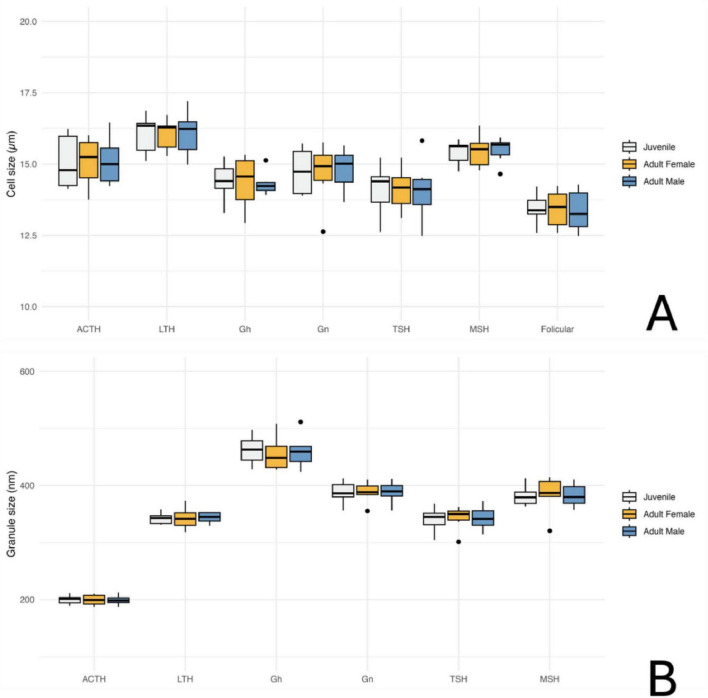
Quantitative analysis of adenohypophyseal cell and granule size in orcas (*Orcinus orca*) using Transmission Electron Microscopy (TEM); **(A)** Average cell size of different hormone-producing cell types in the adenohypophysis, categorized by age and sex. ACTH, corticotrophs; LTH, lactotrophs; GH, somatotrophs; Gn, gonadotrophs; TSH, thyrotrophs; MSH, melanotrophs. **(B)** Average size of secretory granules in the same adenohypophyseal cell types, also categorized by age and sex. Measurements are based on ultrastructural observations under TEM.

Transmission electron microscopy revealed that the adenohypophysis of orcas is composed of a heterogeneous population of endocrine cells, distinguishable by differences in granule morphology, cytoplasmic organization, and intracellular components. Although preservation was not optimal in all samples, six hormone-secreting cell types were identifiable based on general ultrastructural features (Figure 7) and one non-granular cell type was also identified (Figure 8). To complement the morphological classification, quantitative comparisons of cell sizes across age and sex groups (Figure 9A) and secretory granule diameters among the granular cell types (Figure 9B) were performed. These measurements further supported the distinction between cell populations and suggested potential variations in cell structure related to biological variables such as age and sex.

#### 3.4.1 Corticotrophs

The corticotrophs were medium-sized cells with spherical electron-dense granules, typically located near the plasma membrane and in proximity to fenestrated capillaries (Figure 7A). They presented an average cell diameter of 15.08 μm in adult females, 15.06 μm in juvenile females, and 15.10 μm in males. The granules had an average diameter of 199.36 nm in adult females, 199.86 nm in the juvenile female, and 199.05 nm in the adult male, reflecting their specialized function in hormone secretion, and qualitatively they appeared more abundant in the adult male adenohypophysis. These cells were distributed in groups or clusters.

#### 3.4.2 Lactotrophs

Lactotrophs were the largest endocrine cells observed. They presented an irregular cellular morphology, with very pleomorphic secretory granules of varying sizes and densities (Figure 7B). Their average cell diameter was 16.05 μm in adult females, 16.05 μm in juvenile females, and 16.08 μm in adult males. The granules, although very variable in size, reflecting their dynamic secretory activity nature, had an average diameter of 342.76 nm in adult females, 342.42 nm in juvenile females, and 343.62 nm in adult males.

#### 3.4.3 Somatotrophs

Somatotrophs were relatively small, and displayed dense, spherical granules concentrated toward one pole of the cell (Figure 7C). They displayed a very large nucleus with homogeneous chromatin and evident rough endoplasmic reticulum. The average cell diameter was 14.36 μm in adult females, 14.40 μm in juvenile females, and 14.32 μm in adult males. Their granules, however, were notably the largest granules among all identified endocrine cell types, with an average diameter of 455.74 nm in adult females, 462.31 nm in juvenile females, and 460.22 nm in adult males. The dense packing of granules and the highly organized cytoplasmic structure suggest an efficient secretory process, particularly near sinusoidal endothelium.

#### 3.4.4 Gonadotrophs

Gonadotrophs were relatively large and angular, with medium-sized granules distributed throughout the cytoplasm (Figure 7D). The average cell diameter was 14.66 μm in adult females, 14.75 μm in juvenile females, and 14.82 μm in adult males. The granules had an average diameter of 387.88 nm in adult females, 387.63 nm in juvenile females, and 388.17 nm in adult males, with a more diffuse granule distribution supporting the cell’s role in hormone secretion.

#### 3.4.5 Thyrotrophs

Thyrotrophs were smaller in comparison to other endocrine cell types, with a compact cytoplasm containing small, dense granules (Figure 7E). Their nucleus, however, was the biggest proportionally to the cell size, taking up a great part of the cytoplasm. Their average cell diameter was 14.13 μm in adult females, 14.11 μm in juvenile females, and 14.08 μm in adult males. The granules had an average diameter of 342.70 nm in adult females, 340.43 nm in juvenile females, and 339.67 nm in adult males.

#### 3.4.6 Melanotrophs

Melanotroph-like cells were mainly dispersed throughout the gland, rarely organized into cords. They exhibited granules with variable electron density, some with irregular or scalloped membranes (Figure 7F). These cells presented an average cell diameter of 15.47 μm in adult females, 15.41 μm in juvenile females, and 15.49 μm in adult males. The granules displayed a range of sizes, with an average diameter of 383.57 μm in adult females, 381.84 μm in juvenile females, and 382.72 μm in adult males, reflecting the diverse functional roles of these cells within the endocrine system.

#### 3.4.7 Follicular cells

Follicular cells lacked secretory granules and were distinguished by their elongated, branching cytoplasmic extensions, which formed a supportive network between endocrine cells. They also presented a spherical and central nucleus. These cells formed follicular cavities by close intercellular contact (Figure 8). These cells were smaller in size, with an averaging diameter of 13.43 μm in adult females, 13.44 μm in juvenile females, and 13.36 μm in adult males. Their lack of granules and specialized morphology highlight their supportive function within the hypophyseal structure.

## 4 Discussion

This foundational characterization of the orca pituitary gland enriches the field of marine mammal neuroanatomy and significantly advances our understanding of cetacean endocrinology. By combining gross anatomical, histological, immunohistochemical, and ultrastructural analyses, this study provides a the first uniquely detailed view of the cellular and functional architecture of the hypophysis in orcas. While sharing core organizational features with other cetaceans and mammals, the gland exhibits species-specific traits, such as its elongated morphology, prominent *pars tuberalis*, and complex neuroendocrine interface, that likely reflect the unique ecological and physiological adaptations of orcas. Moreover, in the context of growing attention to cetacean welfare and stress physiology, these results underscore the value of the pituitary gland not only as an anatomical organ but as a potential dynamic indicator of endocrine status and environmental responsiveness (Ganella et al., 2015). In particular, the integration of ACTH localization with cellular morphology and glandular architecture offers a promising framework for evaluating the function of the hypothalamic–pituitary–adrenal (HPA) axis, which governs vertebrate stress responses (Mormède et al., 2007; Herman et al., 2016). Given the ongoing concern for the welfare of captive cetaceans, especially large, socially complex species like orcas, our findings may contribute to the development of biomarkers to assess chronic stress and physiological burden. This study thus lays the groundwork for advancing the science of cetacean welfare and opens new avenues for identifying endocrine indicators applicable to animals under managed care.

At the same time, several limitations must be acknowledged. The small sample size and exclusive focus on under human care individuals restrict the generalized applicability of our findings. However, access to freshly collected, well-preserved pituitary glands from orcas is extraordinarily rare, and opportunities for such detailed analysis in free-ranging animals are extremely limited. In this context, the availability of high-quality postmortem material from managed-care individuals provides an important, otherwise unattainable window into the species’ neuroendocrine anatomy. Future research incorporating samples from wild individuals would be highly valuable to assess the extent of ecological or physiological variability, but until such data are available, studies like ours remain essential for building a foundational understanding.

These findings also demonstrate that detailed postmortem analysis of the pituitary can yield biologically meaningful information about an animal’s history and health, contributing to both basic research and applied welfare or conservation efforts. As threats to orcas continue to intensify—from anthropogenic disturbance to environmental degradation—comprehensive physiological markers, including endocrine system assessments, will be essential for monitoring individual and population-level health. This study contributes novel data across multiple structural levels, contextualized within both comparative anatomy and stress biology, providing a valuable reference for future interdisciplinary research. By emphasizing the importance of species-specific data, these findings lay the groundwork for understanding cetacean adaptation, wellbeing, and long-term survival, while advancing in the fields of endocrinology, welfare, and comparative neurobiology.

### 4.1 Anatomical organization and histological architecture of the pituitary gland of orcas

The overall architecture of the orca hypophysis adheres to the classical mammalian organization (Gorbman, 1941; Betchaku and Douglas, 1981; Lechan and Toni, 2000; Tziaferi and Dattani, 2018), with a clearly delineated adenohypophysis and neurohypophysis. However, it also exhibits particularities shared only with other cetacean species, and species-specific traits reflecting the unique anatomical and physiological adaptations of orcas. Extraction of the gland in continuity with the brain, following previously described protocols (Alonso-Almorox et al., 2025), ensured the preservation of both the whole gland and its associated structures. Notably, the sphenoid bone in orcas was exceptionally thick, necessitating the use of appropriate dissection tools to facilitate ventral access via the basisphenoid, as outlined in the protocol.

Gross anatomical examination revealed a distinctly flattened and elongated gland, embedded within a thick, fibrous, and highly vascularized connective tissue pad. This flattened morphology and robust vascular bed contrast with findings in smaller delphinids, which typically exhibit a more globular gland and a proportionally thinner vascular cushion (Pilleri, 1969; Yablokov et al., 1972; Cozzi et al., 2016). This morphology is aligned with the previous the findings observed through magnetic resonance imaging (MRI) in a 12 year-old male orca, where the adenohypophysis appeared to be independent and trifolded the neurohypophysis in size, and showed a very characteristic flat and elongated shape (Wright et al., 2017). These morphological differences with other cetaceans may reflect cranial constraints in large delphinids or represent functional adaptations for enhanced vascular integration (Cowan et al., 2008; Vuković et al., 2011; Panin et al., 2013; Alonso-Almorox et al., 2025).

The thick, highly vascularized fibrous pad observed surrounding the gland in orcas closely resembles the rete cushion previously described in dolphins, both in gross appearance and histological architecture (Lillie et al., 2022; Alonso-Almorox et al., 2025). Although this structure has not been fully characterized functionally in cetaceans, its gross configuration is consistent with the definition of a rete mirabile, where a single artery branches into a dense network of smaller vessels before converging again (Cozzi et al., 2016). In terrestrial artiodactyls, such retia are often involved in thermoregulatory processes and countercurrent heat exchange, playing a role in protecting sensitive neural tissues from thermal fluctuations (Mitchell and Lust, 2008). The dense vascular network observed in our study may similarly contribute to regulating the temperature and perfusion of the pituitary gland, which is critical for its endocrine function. While direct physiological measurements are lacking, the morphological prominence of this rete-like structure in orcas—especially in comparison to smaller odontocetes—may suggest a possible adaptive role in supporting the metabolic demands of larger brains or in mitigating pressure-related stress during deep dives. Further studies integrating anatomical, physiological, and imaging data are needed to clarify the function and evolutionary significance of this vascular specialization.

*In situ*, the ventral aspect of the adenohypophysis constituted the most visible portion of the gland, while the neurohypophysis was positioned dorsally and slightly caudally, obscured by a thick connective tissue septum. Moreover, both the darker coloration in all orca adenohypophysis examined, in contrast to the white to yellow appearance in dolphin species; and the elongated infundibular stalk, resulting from the extended *pars distalis* reaching toward the hypothalamus, contrast with the shorter stalks observed in smaller odontocetes such as the bottlenose and common dolphins, appear to be unique to orcas and may reflect a vascular and morphological adaptation in these species (Alonso-Almorox et al., 2025).

These anatomical distinctions, while requiring validation with larger sample sizes and more diverse age and sex classes, may reflect ecological, or physiological divergence within the Delphinidae, and emphasize the necessity of developing species-specific reference data for large odontocetes, as their endocrine architecture may diverge from that of smaller cetaceans due to variations in skull morphology, brain positioning, and vascular organization.

### 4.2 Histological architecture of the pituitary gland of orcas

Histological analysis of the orca pituitary revealed a well-organized, highly vascularized gland, enclosed within a robust and similarly vascularized dural capsule. A prominent dural recess—composed of dense connective tissue—clearly demarcated the adenohypophysis from the neurohypophysis, as is characteristic in other studied cetaceans (Wislocki and Geiling, 1936; Vuković et al., 2011).

Within the adenohypophysis, hormone-producing cells were arranged in classical cords, clusters, and ribbons, embedded in a dense capillary network. The pars distalis exhibited high cellularity, with clear histochemical distinction between chromophilic and chromophobic cell types, indicative of functional diversity; and the neurohypophysis or pars nervosa also showed the expected sparse cellularity, consisting primarily of unmyelinated axons, pituicytes, and a dense capillary plexus, both as previously noted in dolphin species (Fair et al., 2011; Suzuki et al., 2018; Alonso-Almorox et al., 2025). In contrast, the pars tuberalis appeared more developed in orcas, composed of elongated cords of endocrine cells often closely associated with vasculature. This region featured a striking neuroendocrine interface not yet reported in other studies, with digitiform histologically distinct interdigitations between neural (infundibular) and endocrine tissues, suggesting enhanced neurovascular integration and efficient hormonal exchange.

### 4.3 Immunohistochemical characterization of adenohypophyseal cells

Immunohistochemical analysis of the orca pituitary revealed distinct regional expression patterns for ACTH, MSH, and TSH, supporting functional compartmentalization within the adenohypophysis. These hormones were selected for their involvement in major neuroendocrine axes and their relevance to critical physiological functions in marine mammals. ACTH is a central effector of the hypothalamic-pituitary-adrenal (HPA) axis and a well-established marker of stress-related activity (Kurusomi and Kobayashi, 1966; Hauger and Dautzenberg, 2000). MSH, derived from the same POMC precursor as ACTH, plays roles in energy balance, appetite regulation, and environmental adaptation, and its cellular localization may shed light on the debated presence or absence of the pars intermedia in cetaceans (Panin et al., 2013). TSH was included due to its essential function within the hypothalamic-pituitary-thyroid (HPT) axis, regulating thyroid hormone production (T3 and T4), which are vital for metabolism, thermoregulation, and growth. Moreover, thyroid disruption has emerged as a critical area of investigation in orcas and other marine mammals due to the bioaccumulation of endocrine-disrupting pollutants—such as persistent organic pollutants (POPs)—that interfere with thyroid hormone homeostasis and have been linked to metabolic, developmental, and reproductive impairments (Tanabe, 2002; Bennett et al., 2021; Bjørneset et al., 2023). The spatial distribution of immunoreactive cells aligned with the classical subdivisions of the *pars distalis* and *pars tuberalis*, as seen in other mammals and odontocetes (Cowan and Gatalica, 2002; Cowan et al., 2008; Alonso-Almorox et al., 2025), indicating a conserved endocrine topography across species.

ACTH-positive cells were the most abundant and showed robust staining across all specimens. Their prominent presence in both the *pars distalis* and *tuberalis*—especially near vascular sinusoids—highlights their key role in the hypothalamic-pituitary-adrenal (HPA) axis (Yoshitomi et al., 2016). The adult male specimen exhibited a higher density of ACTH-immunoreactive cells, potentially indicating sex-related differences in corticotroph activity or stress-related hormonal demands. Sexual dimorphism in glucocorticoid physiology and corticotroph responsiveness has been reported in other mammals, including mice and humans (Kroon et al., 2020; Moisan, 2021; Duncan et al., 2023). This individual was also the oldest among the studied (29 years old), and studies in humans have demonstrated that the volume density of ACTH-producing cells tends to increase with aging (Pavlović et al., 2021). Additionally, animal models have shown similar increases in corticotroph labeling under chronic stress conditions (Kapitonova et al., 2010). Given that these orcas were captive individuals of varying ages and medical histories—including some with documented chronic pathologies—these factors may have contributed to interindividual variability in ACTH expression. While our sample size limits definitive conclusions, the combined effects of age, stress, and health status should be considered when interpreting corticotroph distribution.

MSH-expressing cells were widely distributed, primarily within the *pars distalis*, where they formed small clusters or linear arrangements consistent with patterns reported in other delphinids (Panin et al., 2013; Alonso-Almorox et al., 2025). Notably, these cells were more frequently observed in the dorsal region of the *pars distalis*, adjacent to the dural recess that separates the two hypophyseal lobes, while fewer were detected in the *pars tuberalis*. This distribution pattern is of particular interest because in many mammalian species, MSH-producing cells (melanotrophs) are primarily localized in the *pars intermedia*, a distinct zone juxtaposed between the adenohypophysis and neurohypophysis (Naik, 1973; Takeuchi, 2001). However, the presence of a true *pars intermedia* in cetaceans has long been debated. Most studies suggest that cetaceans lack a distinct *pars intermedia*, with melanotrophs instead dispersed throughout the adenohypophysis, and the dural recess clearly separates the anterior and posterior hypophyseal lobes (Wislocki and Geiling, 1936; Slijper, 1962; Harrison, 1969; Arvy, 1971; Panin et al., 2013; Alonso-Almorox et al., 2025). A similar condition has been reported in other species like manatees, which also appear to lack this intermediate zone (Quiring and Harlan, 1953). However, some authors have described the presence of a so-called “dorsal shoulder” in dolphin species, a distinct region located dorsally to the *pars distalis* and adjacent to the dural septum, characterized by a different cellular organization and arrangement of cell cords. This area has been interpreted as a possible remnant of the *pars intermedia* (Cowan et al., 2008), raising questions about the evolutionary and functional significance of this anatomical variation in cetaceans. The predominance of MSH-expressing cells in the dorsal *pars distalis* of orcas, adjacent to the dural recess, reinforces the hypothesis of a functionally distinct dorsal region in cetaceans and underscores the need for further investigation into the evolutionary and physiological implications of this potential pars intermedia remnant.

TSH-positive cells were the least numerous, localized mainly in the *pars distalis*. Their compact, polygonal shape and discrete staining suggest a tightly controlled secretory function, reflecting the thyroid axis’s sensitivity to metabolic feedback (Hoermann et al., 2015). Despite their low abundance, their proximity to vascular channels likely facilitates effective hormone release. In cetaceans, the hypothalamic-pituitary-thyroid (HPT) axis plays a pivotal role in maintaining metabolic homeostasis amidst the physiological challenges of an aquatic environment, such as thermoregulation or energy balance (Fair et al., 2011; Suzuki et al., 2018). Given these unique adaptations, the structure and function of the cetacean HPT axis deserves further investigation to better understand its role in marine mammal endocrinology and environmental resilience.

Beyond the ACTH-, MSH-, and TSH-immunoreactive cells analyzed, the adenohypophysis comprises additional hormone-producing cell populations that were not targeted in this study but are known to play key physiological roles in mammals- Cells producing growth hormone (GH) contribute to somatic growth, protein synthesis, and metabolic regulation; GH may be particularly relevant for supporting blubber deposition, tissue maintenance, fasting and diving or other metabolic adaptations in cetaceans (Weber et al., 1978; Hemming et al., 1986). Prolactin (PRL)-producing cells are involved in lactation, but also play roles in osmoregulation, immune function, and behavioral modulation, with potential relevance in highly complex social species like orcas. Cells that secrete follicle-stimulating hormone (FSH) and luteinizing hormone (LH) regulate reproductive function and are influenced by both internal and environmental cues, including stress, nutritional status, and contaminant exposure (Hart et al., 1984; Ottinger et al., 2009; Delbes et al., 2022). While the present study focused on a selected set of hormones to provide a first morphological and immunohistochemical framework for orca adenohypophyseal organization, further work is needed to identify and characterize the full range of hormone-secreting cell types in this species. Future studies employing broader immunolabelling approaches will be essential to develop a more complete understanding of the endocrine architecture of the cetacean pituitary and its functional significance in the context of marine mammal physiology.

Together, these findings confirm a structurally and functionally specialized adenohypophysis in orcas, with distinct endocrine cell populations contributing to systemic homeostasis. The immunohistochemical profiles reported here, documented for the first time in this species, generally mirror those observed in other odontocetes, reinforcing the conserved nature of pituitary architecture across body sizes and ecological contexts (Cowan et al., 2008; Alonso-Almorox et al., 2025). While also revealing unique, species-specific features that may reflect the particular physiological demands of this apex marine predator.

### 4.4 Ultrastructural characterization of endocrine cell types

Transmission electron microscopy revealed multiple endocrine cell types in the adenohypophysis of orcas, distinguishable by differences in size, granule morphology, and cytoplasmic organization. Although ultrastructural preservation was suboptimal in some specimens, limiting detailed resolution and requiring broader morphological descriptions compared to prior odontocete studies (Young and Harrison, 1970, 1977; Alonso-Almorox et al., 2025), the overall cell types and architecture aligned with those described in mammals, allowing clear cellular identification (Moriarty, 1973; Uei and Kanzaki, 1976; García-Ayala et al., 1997). The small sample size, a consequence of limited postmortem access to orcas, also constrained statistical comparisons, especially for sex- or age-related variation. Quantitative estimation of the relative abundance of different endocrine cell types was not feasible due to both the limited number of samples and inconsistent tissue preservation. Moreover, our study focused on the endocrine aspects of this gland, leaving the neurohypophysis ultrastructurally unexplored. Future research should include the *pars nervosa* as well, to obtain a fuller perspective of the neuroendocrine nature of this gland. Nonetheless, these findings offer a valuable reference for future neuroendocrine and welfare studies in this species and enrich broader knowledge on cetacean pituitary complexity.

The spatial distribution and organization of endocrine cells were comparable between orcas and smaller odontocetes like common dolphins (Young and Harrison, 1970, 1977; Alonso-Almorox et al., 2025). Both showed marked cellular pleomorphism, with secretory cells arranged singly or in clusters, often near fenestrated capillaries—facilitating hormone release, as expected in vascularized glands. The parenchymal structure, including a delicate reticulin network, and the identification of well-developed rough endoplasmic reticulum and Golgi complex in the endocrine cells were also similar across species, underscoring conserved roles in protein synthesis and granule formation, though ultrastructural assessment in orcas was hindered by preservation quality.

Lactotrophs showed pleomorphic morphology, with heterogeneous granules and prominent Golgi zones, consistent with their lactogenic function as seen in other mammals (Hemming et al., 1986; Christian et al., 2015). In orcas, in opposition to what was observed in common dolphins, lactotrophs were the largest endocrine cells, while somatotrophs, on the other hand, were relatively small cells, featuring the largest secretion granules of the adenohypophysis, supporting their role in growth hormone production (Alonso-Almorox et al., 2025). Among non-granular cells previously described in dolphins, only follicular cells were observed. These lacked granules and displayed elongated projections forming intercellular scaffolds and cavities, with proportions comparable to dolphins. Capsular cells were not identified, possibly due to tissue degradation, but further studies are needed to confirm their presence.

Corticotrophs were identifiable by their small, spherical, electron-dense granules adjacent to plasma membranes and capillaries. Their cytoplasmic profile suggested active ACTH secretion, consistent with the observed strong immunohistochemical labeling. A greater abundance of secretory granules was qualitatively noted in the adult individuals compared to the juvenile, with the adult male exhibiting the highest proportion of granules per cell, indicative of heightened ACTH synthesis or release. These findings align with the results in common dolphins (Alonso-Almorox et al., 2025), and likely reflect differing hormonal demands for stress and energy regulation (Naik, 1973; Uei and Kanzaki, 1976; Duncan et al., 2023). The pronounced granule density in the adult male may reflect multiple contributing factors. As the oldest individual (29 years), age-related changes in corticotroph function are likely, as aging has been associated with increased ACTH cell activity (Pavlović et al., 2021). His documented chronic pathology may have further stimulated the HPA axis, given that prolonged stress and illness are known to affect corticotroph dynamics (Kapitonova et al., 2010; Herman et al., 2016). While such factors are common in captive settings, similar health-related biases also affect studies of stranded or free-ranging individuals. Additionally, the higher granule content in the male may relate to sex-linked differences in corticotroph function and glucocorticoid metabolism, as reported in other mammals (Kroon et al., 2020; Moisan, 2021; Duncan et al., 2023). These findings, together with immunohistochemical data, highlight a multifactorial modulation of corticotroph morphology and physiology. Understanding this complexity is essential for accurately evaluating the welfare and physiological state of cetaceans, especially given the central role of the HPA axis in mediating responses to environmental and internal challenges.

This study provides the first integrative anatomical, histological, and ultrastructural description of the orca pituitary gland, establishing a robust baseline for future neuroendocrinological research. By revealing both conserved and species-specific features, our findings deepen the understanding of cetacean neuroendocrinology and highlight the importance of species-specific reference data. The pituitary emerges as a sensitive indicator of endocrine function, stress physiology, and overall health status. As anthropogenic pressures continue to mount, interdisciplinary efforts such as this one are essential for informing welfare assessments and guiding conservation strategies in large, long-lived marine mammals like orcas. This foundational work opens critical pathways for future studies linking endocrine architecture to environmental, physiological, and behavioral variables in both managed and wild cetacean populations.

## Data Availability

The original contributions presented in this study are included in this article/Supplementary material, further inquiries can be directed to the corresponding author.
