# Supplementary material for: The orca (Orcinus orca) pituitary gland: an anatomical, immunohistochemical and ultrastructural analysis
Source: Front Neuroanat. 2025 Jul 17;19:1626079. doi: 10.3389/fnana.2025.1626079 (PMC12310606; doi:10.3389/fnana.2025.1626079)
Supplement: Supplementary file 1 [file Data_Sheet_1.docx]

Supplementary Material

| **Antibody (Target)** | **RRID** | **Target Protein (Species)** | **Orca Protein Accession** | **Query Cover** | **% Identity** | **Conclusion** |
| --- | --- | --- | --- | --- | --- | --- |
| Anti-ACTH (POMC) | AB_1158079 | Human POMC (138–150) | XP_004268145.1 | 100% | 100.0% | Full match; strong cross-reactivity expected |
| Anti-MSH (POMC) | AB_260462 | Human POMC (MSH region) | XP_004268145.1 | 100% | 100.0% | Full match; strong cross-reactivity expected |
| Anti-TSHβ | AB_1158910 | Human TSHB | XP_033291983.1 | 100% | 89.13% | High similarity; cross-reactivity likely |

Supplementary Table S1. BLASTp validation of primary antibodies used in this study against *Orcinus orca* protein targets. For each antibody, the target protein sequence from the species of original reactivity was compared to the corresponding *Orcinus orca* protein using NCBI BLASTp. The table includes antibody name and target, Research Resource Identifier (RRID), source species of the target protein, the corresponding *O.orca* protein accession number, alignment metrics (query coverage and percent identity), and a conclusion on the likelihood of cross-reactivity. Full or high sequence identity supports the suitability of these antibodies for immunohistochemical applications in *Orcinus orca* tissues.


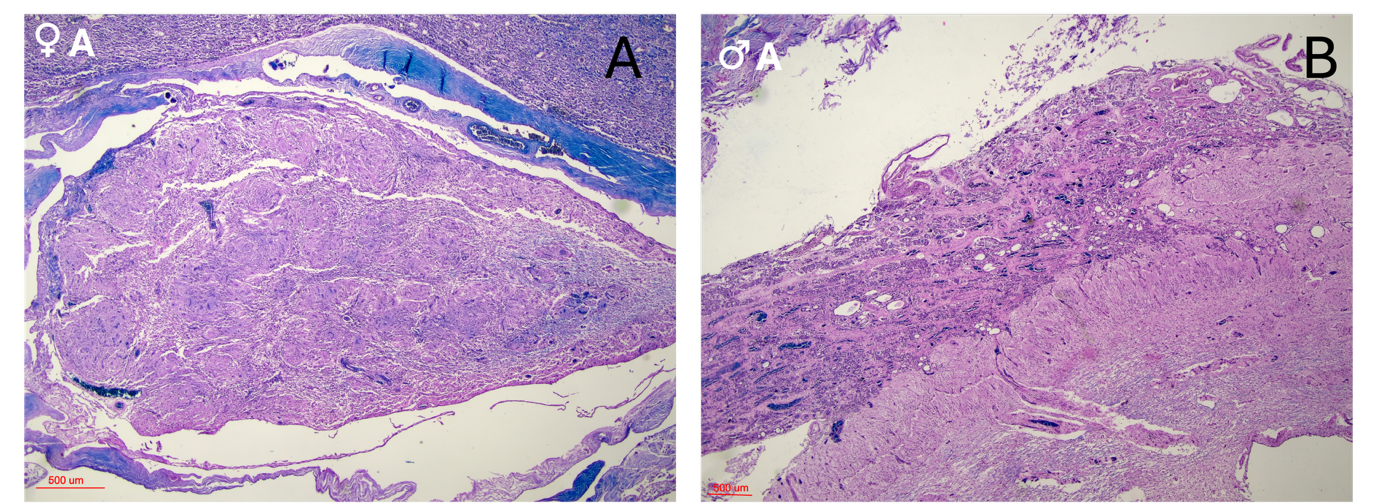


Supplementary Figure 1. Histochemical characterization of the neurohypophysis and infundibular neuroendocrine connection in *Orcinus orca*, using Luxol fast blue to evaluate myelination patterns. The absence of Luxol Fast Blue staining confirms the non-myelinated profile of these neuroendocrine pathways, consistent with their role in neurohormone release rather than rapid signal transmission.(A) Neurohypophysis of an adult female, stained at 2× magnification, shows the predominance of unmyelinated nerve fibers within the *pars nervosa* (neurohypophysis); (B) Infundibular region of an adult male, also at 2× magnification, further showing the unmyelinated neuroendocrine connection.


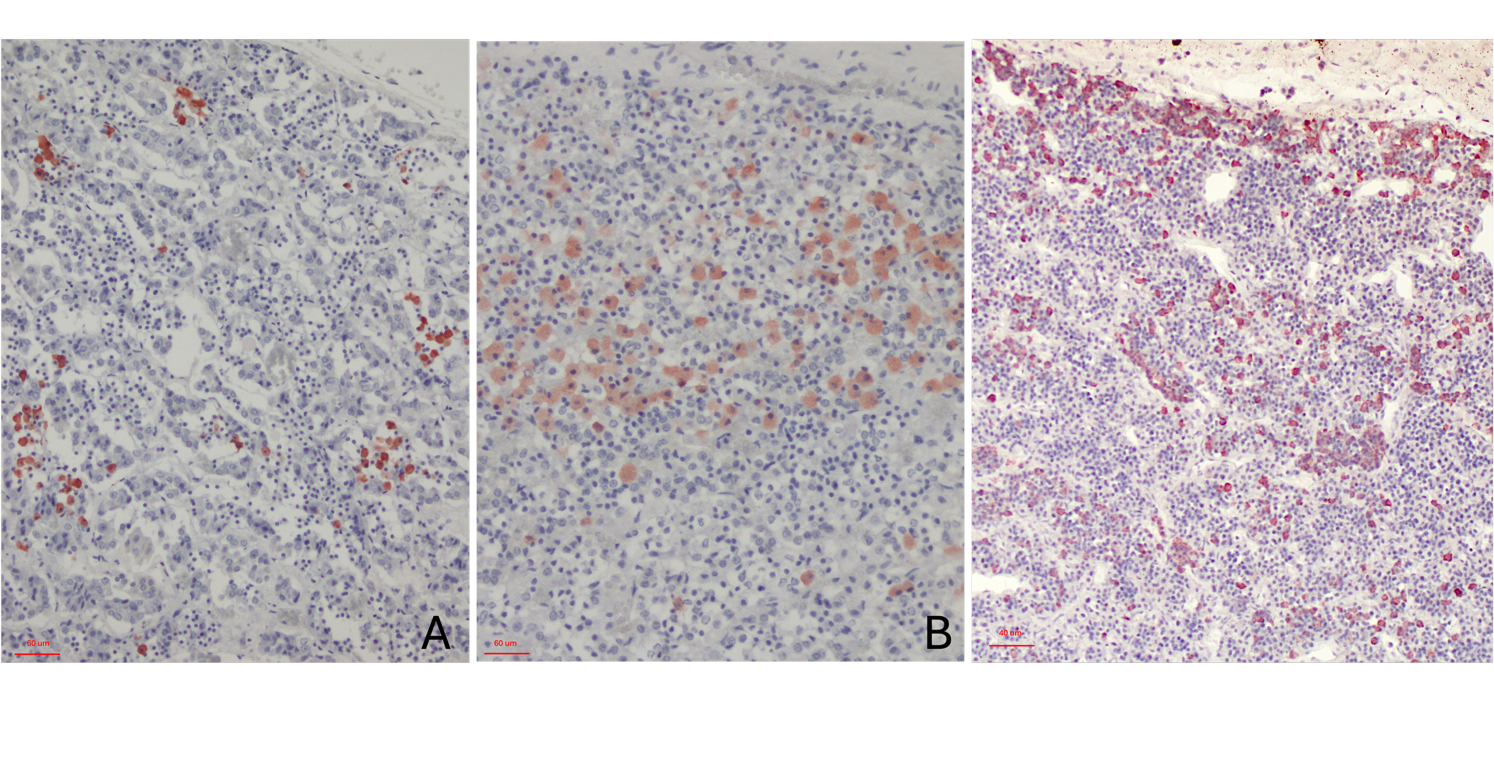


Supplementary Figure 2**.** Comparative immunohistochemical characterization of adenohypophyseal cell populations in an adult male *Orcinus orca*, highlighting the differential spatial distribution and density of endocrine cell types within the adenohypophysis. Serial sections of the same histological level—at the dorsal edge of the adenohypophysis adjacent to the dural capsule—were stained with three different primary antibodies targeting distinct pituitary hormones.(A) TSH immunoreactivity revealed a sparse population of small, scattered immunopositive cells; (B) MSH staining showed a more homogeneous and moderately abundant population, with immunoreactive cells arranged along the dorsal shoulder of the adenohypophysis; (C) ACTH immunoreactivity was markedly more widespread, with dense populations of immunopositive cells distributed in rosettes, clusters, or dispersed throughout the section.


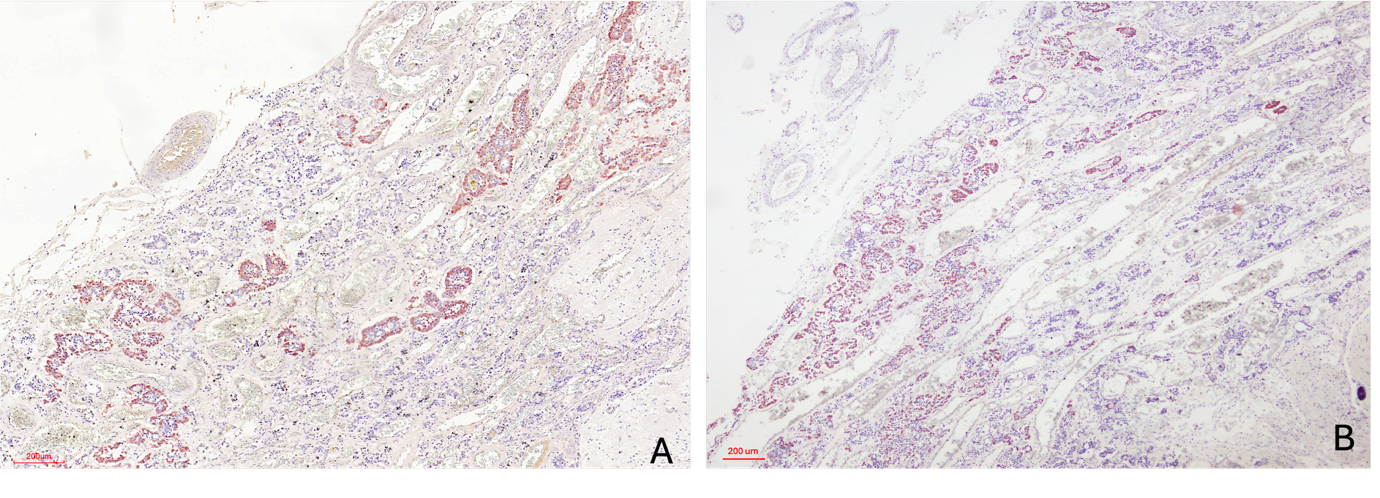


Supplementary Figure 3. Immunohistochemical characterization of adenohypophyseal cell populations in an adult female *Orcinus orca*, at the level of the infundibulum and neuroendocrine connection. (A) MSH immunoreactivity reveals a highly organized distribution pattern, with immunopositive cells arranged in well-demarcated, rosette-like clusters; (B) ACTH staining shows a markedly denser and more heterogeneous distribution, with immunoreactive cells appearing in rosettes, cords, small groups, or individually dispersed throughout the section.
